# MgO and Zn/MgO Nanoparticles as Direct Antifungal Compounds and Elicitors Against *Sclerotinia sclerotiorum* in Pea

**DOI:** 10.3390/jof12040256

**Published:** 2026-04-01

**Authors:** Hanaa Omar, Ahmed Mohamed, Nehad El-Gammal, Warda Hussain, Saleh Alhewairini, Mahmoud Abdelfatah, Abdelhamed Elshaer, Reda Omara, Ayman Omar, Aly Derbalah

**Affiliations:** 1 Genetics Department, Faculty of Agriculture, Cairo University, Giza 12613, Egypt; 2 Plant Pathology Research Institute, Agriclutural Research Centre, Giza 12619, Egypt; 3 Department of Plant Protection, College of Agriculture and Food, Qassim University, Buraidah 51452, Saudi Arabia; a.mohmed@qu.edu.sa; 4 Physics Department, Faculty of Science, Kafrelsheikh University, Kafrelsheikh 33516, Egypt; 5 Pesticides Chemistry and Toxicology Department, Faculty of Agriculture, Kafrelsheikh University, Kafrelsheikh 33516, Egypt

**Keywords:** pea, nanoparticles, white mold, systemic acquired resistance, resistance induction, pathogenesis-related genes

## Abstract

The purpose of this study was to assess the effectiveness of two nanostructures (MgO and Zn/MgO) against *Sclerotinia sclerotiorum*, which causes white mold disease in peas, as direct antifungal agents or resistance inducers in pea plants. The direct antifungal activity of these nanostructures was evaluated by assessing their ability to inhibit *S. sclerotiorum* growth in vitro and reduce white mold severity in the greenhouse. The induction of resistance in pea plants was examined by assessing the expression of three defense-related genes using quantitative real-time PCR and measuring the phenolic compounds content in treated pea plants relative to untreated controls. The effect of the tested control agents on the growth and yield of pea plants was investigated. In comparison to the untreated control, *S. sclerotiorum* growth was markedly suppressed following treatment with the investigated compounds. The complete suppression (100%) of *S. sclerotiorum* growth was achieved with concentration levels of 100 mg/L for both MgO and Zn/MgO nanostructures. In greenhouse conditions, pea plants treated with the investigated chemicals showed a considerable reduction in the severity of white mold disease when compared to the untreated control plants. The transcript levels of 12-oxophytodienoate reductase 11 (*OPR1*), antioxidant peroxide (*PsOXII*), and chlorophyll a-b binding protein genes increased significantly in treated plants with MgO (3.1, 2.7, and 3.5-fold), fungicide (3.2, 2.8, and 2.8-fold), and Zn/MgO (3.5, 3, and 5-fold) compared to control, respectively. Pea plants treated with the tested nanoparticles generated more phenolic content than untreated controls. The application of fungicide and tested nanoparticles to peas greatly enhanced their growth properties. In light of our results, the application of these nanoparticles may represent a novel approach for controlling this pathogen.

## 1. Introduction

The pea, or *Pisum sativum* L., is an annual herbaceous plant belonging to the Leguminosae or Fabaceae family. Grown in numerous locations worldwide, it is a significant and well-liked food crop, particularly for its edible seeds [1]. With a total production of 10.2 million tons, peas rank fourth among all legumes consumed worldwide [2]. According to Bhat et al. [3], peas are valued mainly for their high nutritional content, which includes 23–25% protein [4], 4–10% of the sugars, 20–25% starch, and 0.6–1.5% fat [5]. Furthermore, a number of vital minerals that are typically lacking in cereals, such as calcium, phosphorus, and iron, are abundant in peas [6].

*S. sclerotiorum* (Lib.) de Bary is a global necrotrophic fungal disease that has been shown to affect over 600 species of higher plants [7,8,9]. Sclerotinia outbreaks have been repeatedly recorded in pea crop locations around the world [10,11]. The pathogen affects the stem, leaf, or pod tissue of plants and produces water-soaked lesions with a white cottony mass of mycelium on the surface [12,13]. It is a devastating plant disease, and its pathogen is not only ubiquitous but also has the potential to feed as a hemibiotroph on these plants [14,15]. White mold, caused by *S. sclerotiorum* (Lib.) de Bary, reduces yield in most dicotyledonous crops [8,16,17]. The management of *S. sclerotiorum* is challenging due to the fact that it affects such a wide range of host plants, coupled with long-term survivability and genetic variability [18].

The use of fungicides to manage Sclerotinia aims not only to protect current yields by reducing disease establishment and severity but also to protect future yields by reducing sclerotia accumulation in the soil, thereby preventing the initiation of new infection cycles [19]. However, significant dependence on expensive commercial fungicides, along with repetitive, poorly timed, and often indiscriminate usage, has resulted in the pathogen acquiring an insensitivity to the active ingredient(s), leading to reduced fungicide efficacy in pathogen control. The risk of *S. sclerotiorum* developing resistance to popular fungicides, such as benomyl and carbendazim [20,21], has prompted the search for alternative control strategies that are necessary to manage this disease, limit its spread, and minimize damage. Extensive research has focused on addressing persistent and excessive reliance on conventional chemical pesticides, implying that nanoscale materials may provide a promising alternative solution [22,23,24,25,26,27]. Nanoparticle materials’ unique physicochemical features distinguish them from bulk materials and improve their ability to interact with microbial species and perform various antimicrobial functions [24,25,27,28,29]. Zinc oxide (ZnO) and magnesium oxide (MgO) nanoparticles are potent antibacterial and anti-odor agents [30]. ZnO and MgO nanostructures are attractive antimicrobial ingredients in many products due to their increased ease of dispersibility, optical transparency, and smoothness [26,31].

Plant pathogens can be controlled either directly by the antimicrobial activity control agents or indirectly by enhancing the host’s defense mechanisms and producing an induced systemic resistance (ISR) that strengthens the plants’ capacity to fend off subsequent attacks from various pathogens [24,25,27]. A promising approach for decreasing disease severity relies on the stimulation of systemic acquired resistance (SAR) and involves impacts of protein cross-linking, hydrogen peroxide (HR2ROR2R) accumulation, and peroxidase activity [32,33]. A different approach to using nanoproducts for managing plant diseases through the induction of plant immunity has been proposed. The goal of enabling plants to protect themselves against diseases is to activate defensive response pathways [23,24,25,26,27,34]. Biochemical interactions and stress conditions in plants are directly correlated with the phenolic content produced. Previous studies have shown that during both biotic and abiotic stress, the phenolic content in plants increases significantly [35]. Phenolic compounds are among the most important and widely distributed secondary metabolites in plants, playing a crucial role in disease resistance across various crop species. These compounds are involved in essential physiological processes such as growth, reproduction, pigmentation, rhizogenesis, and pathogen resistance [35,36,37]. Additionally, they contribute to the genetic resistance of plants against pathogen infections [35,38]. The oxidation products of phenolic compounds are also closely linked to the resistance of plants to diseases [35,39].

The purpose of this study was to evaluate the antifungal activity of some nanostructures (MgO and Zn/MgO) against *S. sclerotiorum* under laboratory conditions, to determine the extent of their ability to control white mold disease in pea plants under greenhouse conditions, to evaluate the ability of MgO, fungicide, and Zn/MgO for the induction of defense responses in pea plants by measuring the expression of 12-oxophytodienoate reductase 11 (OPR1), peroxidase (PsOXII), and chlorophyll a-b binding protein C genes as well as the produced free, conjugated, and total phenols in treated pea plants compared to untreated control, and finally to examine the effect of tested nanostructures on some growth characteristics of pea.

## 2. Materials and Methods

### 2.1. Chemicals

The fungicide used for controlling white mold on peas was tolclofos-methyl (20%) + thiram (30%), commercialized under the trade name Rezolex-T 50% WP and manufactured by Kafr El-Zayat Company for Chemicals and Pesticides, Kafr El-Zayat City, Garbia, Egypt.

### 2.2. Fabrication of MgO Nanoparticles

The sol–gel approach was used to create the MgO nanoparticles, as previously reported [40,41]. A standard procedure involved dispersing 0.2 M magnesium nitrate hexahydrate (Mg (NO_3_)_2_.6H_2_O, WINLAB, 99%) in 100 mm of deionized water (DW). After that, a solution of magnesium (NO_3_)_2_.6H_2_O was mixed with 0.4 M sodium hydroxide [NaOH, OXFORD, 99%] by sonication, and the pH was adjusted to 11. According to the equation below, a white gel of Mg (OH)_2_ was created [42]:

Mg (NO_3_)_2_ + 2NaOH → Mg (OH)_2_ + 2NaNO_3_

After filtering and cleaning the resulting Mg (OH)_2_ with deionized water (DW), ethanol was washed to remove any remaining precursors. To eliminate useless water from the sample, it was dried in an oven set at 100 degrees Celsius (°C) for a full day. To create MgO nanoparticles, Mg (OH)_2_ was heated up for five hours at 300 °C using the breakdown equation [42].

Mg (OH)_2_ → MgO + H_2_O

### 2.3. Fabrication of Zn/MgO Nanoparticles

MgO nanoparticles were manufactured by the sol–gel method as previously reported [40,41]. Then, 0.2 M magnesium nitrate hexahydrate [Mg (NO_3_)_2_.6H_2_O, WINLAB Company, Jalgaon, Maharashtra, India, 99%] was dispersed in 100 mL DW. Then 0.4 M sodium hydroxide [NaOH, OXFORD Lab Fine Chem LLP, Palghar, Maharashtra, India, 99%] was added under sonication until the pH was adjusted to 11. 5% weight of zinc nitrate hexahydrate (Zn (NO_3_)_2_.6H_2_O, Sigma-Aldrich, Mumbai, Maharashtra, India, 99%) was finally added to the solution. A white gel of Zn/Mg (OH)_2_ was produced that was calcined to obtain Zn/MgO nanocomposite.

### 2.4. Characterization of Fabricated Nanoparticles

The crystal structures were characterized using X-ray diffraction (XRD, Shimadzu 6000, Shimadzu Corporation, Kyoto, Japan), and the sample morphology was examined using a scanning electron microscope (JEOL (JSMIT100) Hining Tech (Xuzhou) Co., Ltd., Xuzhou, China).

### 2.5. Screening of the Tested Materials Against S. sclerotiorum In Vitro

Under lab settings, the antifungal activity of the two tested nanoparticles (MgO and Zn/MgO) and the recommended fungicide was assessed against *S. sclerotiorum* (Eg-Gh-Ps1 strain), which was isolated from pea and obtained from the Research Institute of Plant Pathology, Agricultural Research Center, Giza Egypt, in slant Agar. A 60 mL volume of autoclaved potato dextrose agar (PDA) that had been cooled to roughly 45 °C was mixed with the appropriate amount of stock solution to obtain the requisite concentrations (20, 40, 60, 80, and 100 ppm) for the tested nanoparticles and 2, 4, 6, 8 and 10 ppm for the fungicide. Nine-centimeter Petri dishes were utilized as replicates for each concentration of every treatment, including the control. The tested compounds were not added to the treatment of the control. A disk (5 mm in diameter) containing the mycelium development from a 5-day-old *S. sclerotiorum* culture was used to inoculate the center of each dish. Parafilm was used to seal the dishes in order to prevent volatile chemicals from evaporating. The dishes were kept at 28 °C until the control treatment’s mycelium reached the plate’s edge. This experiment was repeated three times. Using the formula of Vincent [43], the inhibition % of *S. sclerotiorum* radial growth was determined using the following equation.

I% = A − B/A × 100

where A = the radial growth of the tested fungus in control and B = the radial growth of the tested fungus in treatment

### 2.6. Efficacy of Tested Treatments Under Greenhouse Conditions

For inoculation, stock cultures of the isolated pathogenic fungus *S. sclerotiorum* (Lib) de Bary were grown independently on rice husk medium. Each of the 500 mL glass bottles held 100 g of rice hulls, 200 g of sand, and 100 mL of water, which were autoclaved for 20 min at 1.5 atm. Following that, three mycelial disks (6 mm in diameter) removed from the outermost portion of an actively developing colony of a fungal strain on PDA were added to each bottle of media to inoculate it separately. For seven days, the glass bottles containing the rice culture hulls were incubated at 18–22 ± 2 °C.

The Gemmeiza Agricultural Research Station, Plant Pathology Research Institute, Agricultural Research Center, Giza, Egypt, is home to the greenhouse where this experiment was carried out. After sterilizing with a 5% formalin solution, the sandy-clay soil was compacted into pots of 25 cm in diameter, weighing 4 kg per pot. A week prior to sowing, husk rice culture (*w*/*w*) was artificially infected in pots at a rate of 3%. The inoculated potted soil was kept moist until seeding. Every pot had five seedlings sown.

There were four replicates of each treatment. In a greenhouse, pots were maintained and given water as needed. A 1 g/kg seed of tolclofos-methyl + thiram was applied. Only the pathogenic fungus occupied the control pots. MgO, Zn/MgO (0.080 and 0.100 g ai/kg seeds), and tolclofos-methyl+thiram (1.0 g ai/kg seeds) were applied to pea seeds (var. Master B) by soaking them in the solution for two hours and then leaving them to dry for an hour. Subsequently, the seeds were sown as previously indicated, and thirty days after the plants were sown, the tested nanoparticles were applied to the soil at three separate intervals of ten days, drenching the roots of the seedlings. This experiment was repeated three times.

Pre-emergence damping-off disease incidence was noted 15 days after seeding, whereas post-emergence damping-off and surviving plants were noted 45 days after seeding.

% Pre-emergence damping-off = “No. of non-emerged seedlings”/“No. of sown seeds” × 100

% Post-emergence damping-off = “No. of killed seedlings”/“No. of sown seeds” × 100

% Survival rate = “No. of surviving plants”/“No. of sown seeds” × 100

### 2.7. Plant Defense Genes Expression Using Quantitative Real-Time PCR

To better clarify the regulation and molecular mechanisms of pea plants, their response to *S. sclerotiorum* pathogen infection by activating the synthesis of a diverse number of defense genes (*OPR1*, *PsOXII*), and chlorophyll a-b binding protein) was evaluated using quantitative real-time PCR [44]. RNA was extracted from the leaves of pea plants treated with MgO, fungicide and Zn/MgO collected after 24 h of the last treatment. Samples were directly frozen in liquid nitrogen after harvesting and stored at 80 °C until use. A total of 100 mg of pea plants was extracted using the RNeasy^®^ Plant Mini Kit (Qiagen, Hilden, Germany). RNA concentrations were determined using a NanoDrop ND-100 spectrophotometer, Mettler-Toledo International Inc., Columbus, OH, USA. RNA quality indicators included the 260A ratio. A total of 1 to 2 μg of DNase I-treated RNA was reverse transcribed using the PrimeScript First Strand cDNA Synthesis Kit (Thermo Kit, Thermo Fisher Scientific Baltics, Vilnius, Lithuania). cDNA was estimated by quantitative real-time PCR using the SYBR kit (Takara, Takara Bio, Shiga, Japan) in the Thermal Cycler Bio-Rad Real-Time System II (Bio-Rad, Hercules, CA, USA).

Primers specific for the selected three genes were designed using Primer Blast (https://www.ncbi.nlm.nih.gov/tools/primer-blast/, accessed on 4 June 2025) and Primer3 (https://primer3.ut.ee/, accessed on 4 June 2025) software, as presented in Table 1. Primers were used in RT-qPCR analysis to determine fragments of ~80–200 bp in length using the Takara SYBR^®^ Premix Ex Taq^TM^ (Takara Bio, Shiga, Japan) in 25 μL reactions containing 60 ng template cDNA, 5 μL of 1 μmol/L of each oligonucleotide, and 12.5 μL SYBR premix ExTag. Amplifications were performed by initial denaturation at 95 °C for 120 s followed by 40 cycles at 95 °C for 15 s and at 60 °C for 30 s. Melting curves were examined for each data point to check the specificity of the PCR product. The relative expression levels through the average cycle threshold (CT) were successfully determined. Average CT values were estimated from the triplicate experiment conducted for each gene; the CT value was detected by subtracting the average CT value of genes from the CT value of the Alaska alpha-tubulin gene. Tubulin was used as a housekeeping gene to normalize cDNA concentrations. The relative expression levels of the target genes were calculated using the ΔΔCt method [45] and the reference gene Alaska alpha-tubulin for the treated cells. Finally, a fold change equation (2^−1^) was used to estimate relative expression levels, while the standard deviation was calculated from the replicated experimental data.

### 2.8. Biochemical Analysis

Phenolic content in pea leaves was determined using the colorimetric method as reported by Snell and Snell [46].

#### 2.8.1. Sample Preparation

Crude extracts were produced using 7 g of oven-dried pea leaves in an ASE-200 (Dionex, Thermo Fisher Scientific Baltics UAB, Vilnius, Lithuania) extractor. Extractions were performed with 80% (*v*/*v*) aqueous methanol with shaking for 2 h at room temperature. Extracts were centrifuged at 1000× *g* for 15 min, and 1 mL of each supernatant after filtration was taken to develop the colorimetric reaction [47].

#### 2.8.2. Determination of Total Phenols

The amount of total phenols was ascertained by boiling 0.03 mL of the sample in a water bath for 10 min after adding 0.5 mL of concentrated HCL. Following cooling, 2.0 mL of NaCO_3_ (20%) and 0.5 mL of Folin-Denis reagent were added. Distilled water was added to the mixture to bring the total volume to 10 mL. Using a colorimeter, the color density was measured at 520 nm after 20 min. A standard curve was created for catechol, and the total phenol content was estimated, with results shown as µg catechol/mL of plant extract [48].

#### 2.8.3. Determination of Free Phenols

By mixing 2.0 mL of NaCO_3_ (20%) with 0.5 mL of Folin-Denis reagent (Sigma-Aldrich, Mumbai, Maharashtra, India) and 0.03 mL of the sample, free phenols were determined. After adding distilled water to bring the total volume to 10 mL, it was allowed to stand for 20 min. The color density was measured at 520 nm. Calculating from the catechol standard curve, the amount of free phenols was reported as µg catechol/mL of plant extract [48].

#### 2.8.4. Determination of Conjugated Phenols

Conjugated phenols were calculated by deducting free phenols from total phenols [48].

### 2.9. Statistical Analysis

Greenhouse data were analyzed statistically using a one-way ANOVA test using SPSS software version 15, and after the ANOVA test, the different means were compared using Tukey range tests (*p* ≤ 0.05).

## 3. Results

### 3.1. Characterization of Fabricated MgO

**Figure 1:**
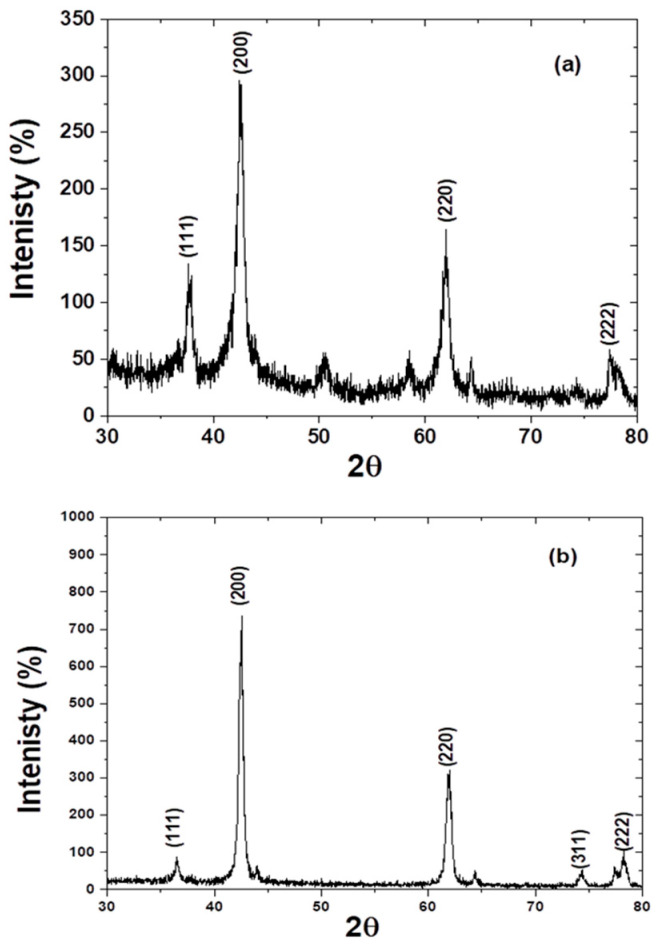
X-Ray diffraction patterns for (**a**) MgO nanoparticles and (**b**) Zn/MgO nanoparticles.

**Figure 2:**
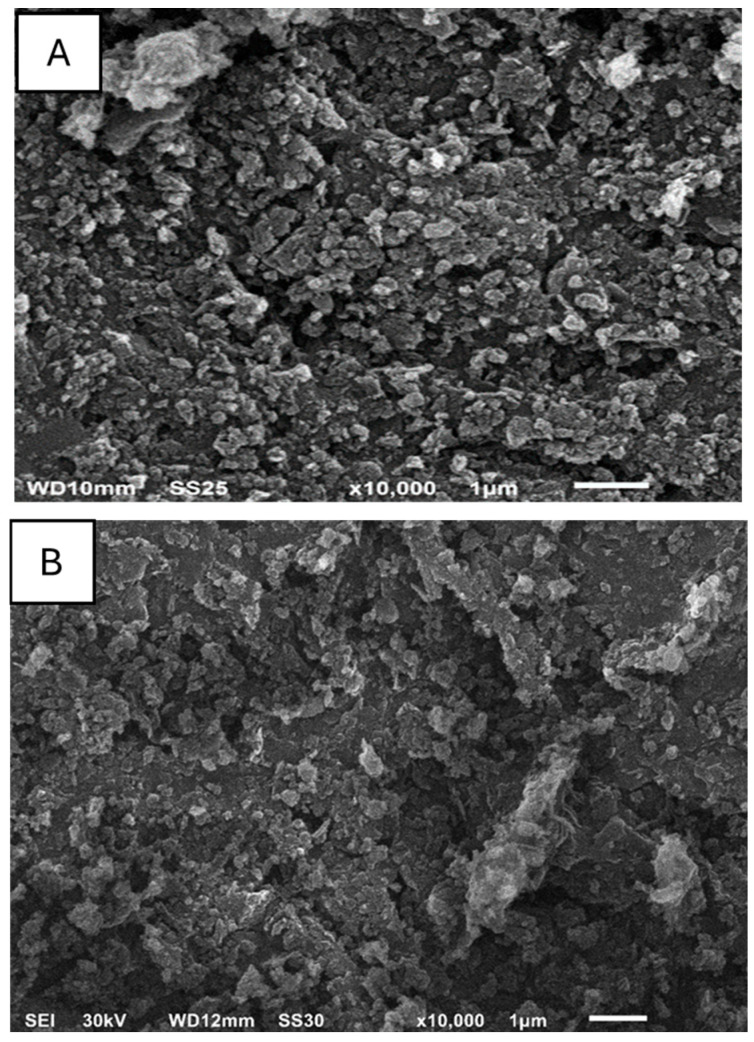
Top-view SEM images of calcined MgO (**A**) and Zn/MgO (**B**) nanoparticles at 10,000× magnification.

The direction planes (111), (200), (220), and (222) that emerged in the XRD patterns of MgO nanoparticles are displayed in Figure 1a. This suggests that MgO nanoparticles are formed on cubic crystal structures [41,49,50]. Furthermore, no peaks for contaminants or other MgO nanoparticle phases were observed. Using the Debye-Scherrer equation, the average particle size was determined:

χ = K (λ/b cos θ)

where λ is the x-ray wavelength produced by Cu Kα1 radiation, which is equal to 0.154 nm, χ is the average particle size, K is the Scherrer constant, which is equal to 0.89, b is the FWHM of the observed peak, and θ is the Bragg angle [50]. It is determined that the average size of MgO nanoparticles is 25 nm. The top-view SEM picture of an aggregation of MgO nanoparticles, which has the spherical forms of homogeneous granules, is shown in Figure 2A. The existence of voids and interstitial spaces implies a highly developed surface structure, indicating an increase in specific surface area and the accessibility of active sites [41].

### 3.2. Characterization of Fabricated Zn/MgO

XRD patterns of Zn/MgO nanocomposite are presented in Figure 1b, where the direction planes (111), (200), (220), (311), and (222) existed, which confirmed the formation of MgO nanoparticles on a cubic crystal structure [41,49,50]. Moreover, no peaks were observed for impurities or other phases of MgO nanoparticles. No peaks appeared for Zn, which means that Zn ions were dispersed inside MgO nanoparticles. The absence of Zn-related diffraction peaks in the XRD pattern of the Zn/MgO sample implies that Zn does not form a separate crystalline ZnO phase but is instead highly dispersed or incorporated into the MgO lattice. Such behavior could be explained by the close similarity between the ionic radii of Zn^2+^ (0.74 Å) and Mg^2+^ (0.72 Å), which permits Zn^2+^ ions to substitute Mg^2+^ ions in the cubic MgO lattice without producing an apparent secondary phase. Such substitutions typically result in minor lattice distortion [41,51]. The observed changes in peak broadening and relative intensities in the Zn/MgO pattern compared to pure MgO further support the modification of the MgO crystal structure by Zn incorporation. The Debye-Scherrer equation was applied to calculate the average particle size, which is found to be 40 nm [41,50].

The top-view SEM picture of an aggregation of MgO nanoparticles, which has the spherical form of homogeneous granules, is shown in Figure 2B [41,52]. Upon Zn incorporation, the Zn/MgO sample is presented in Figure 2B, where a noticeable modification in microstructural features could be observed through denser agglomerates and more compact clusters compared to pristine MgO. The surface becomes obviously more heterogeneous with the presence of elongated and irregular domains, which may relate to Zn-containing species dispersed on the MgO matrix. This morphological transformation suggests a strong interaction between Zn and MgO. The improved roughness of the surface and particle coalescence further confirm the effective loading of Zn and its significant influence on the textural properties of MgO, which is expected to enhance the material’s catalytic and physiochemical performance [41,52]. The spherical shape of MgO and Zn/MgO offers a larger surface area relative to their volume compared to other shapes (like rods or cubes of similar size). This maximized contact area allows for more extensive interaction with the fungal cell wall and membrane, which is the initial step in the antifungal mechanism and facilitates cellular uptake [53].

### 3.3. Antifungal Activity of the Tested Nanoparticles Against S. sclerotiorum Under Laboratory Conditions

**Figure 3:**
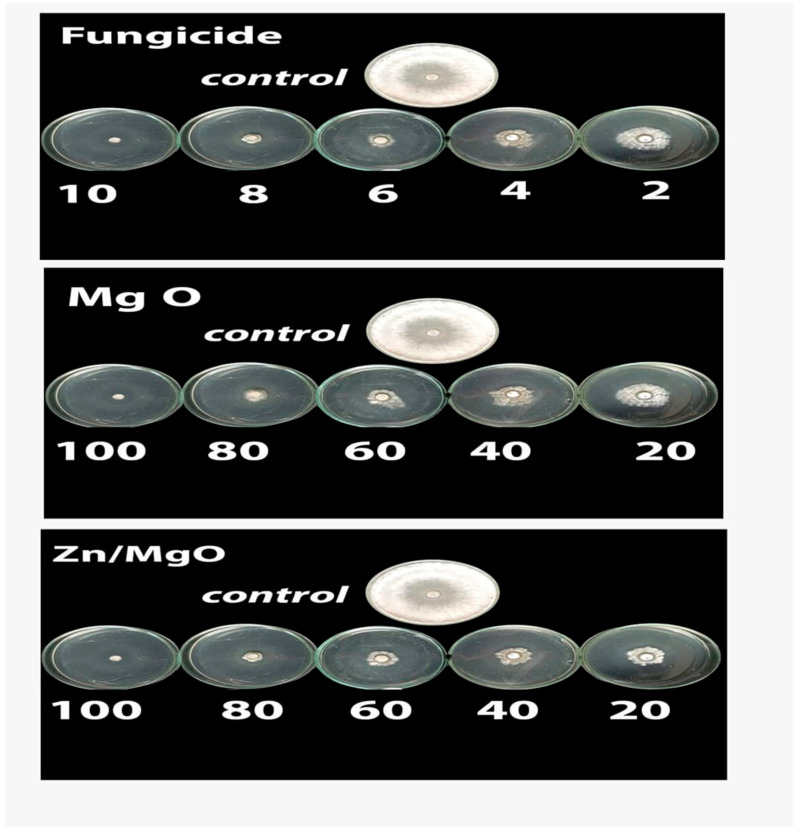
The hyphal growth of *S. sclerotiorum* fungus treated with MgO, Zn/MgO, and fungicide at different concentrations (mg/L) compared to the untreated control.

Table 2 and Figure 3 show the impact of the tested nanostructures and fungicide on *S. sclerotium* mycelial growth in comparison to the control. The findings demonstrated that, in comparison to the untreated control, the application of the tested nanostructures greatly inhibited the growth of *S. sclerotiorum*. Fungicide proved to be the most successful treatment, with Zn/MgO and MgO following closely behind. Furthermore, a strong correlation was observed between the concentrations of the tested nanostructures and their inhibition percentages, with inhibition percentage values of 52, 65, 75, 91, and 100% for MgO and 57, 69, 77, 92, and 100% for Zn/MgO at concentration levels of 20, 40, 60, 80, and 100 mg/L, respectively.

### 3.4. Efficacy of the Tested Control Agents Against White Mold in Peas Under Greenhouse Conditions

Table 3 shows the efficacy of the tested nanoparticles in comparison to the recommended fungicide in terms of pre- and post-emergence, as well as the survival percentage of treated seeds versus untreated control. The results showed that the recommended fungicide was the most effective treatment against white mold, followed by Zn/MgO and MgO in both growing seasons. The efficacy of the nanoparticles studied increased with increasing concentration. Furthermore, the tested nanoparticles performed better in the first season than in the second season.

### 3.5. Expression of Biotic Stress-Related Genes in Treated Pea Plants Relative to Control

**Figure 4:**
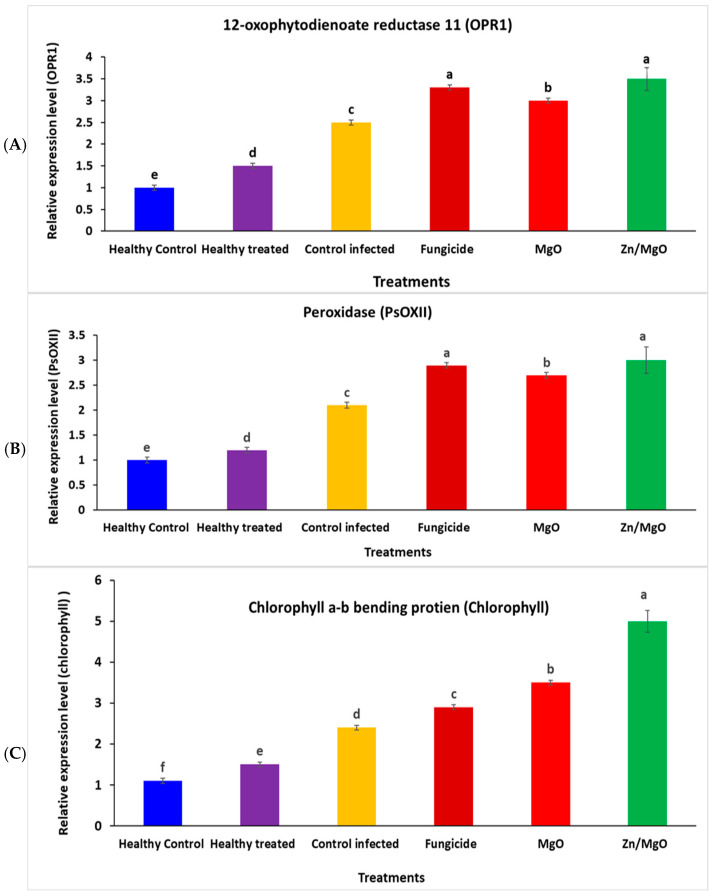
Differential expression of three defense-related genes (**A**) 12-oxophytodienoate reductase 11 (*OPR1*), (**B**) peroxidase (*PsOXII*) antioxidant, (**C**) chlorophyll a-b binding protein C (Chlorophyll) in peas treated with MgO, fungicide, and Zn/MgO compared to untreated control. Different lowercase letters represent significant differences between the treatments for each analyzed gene using the Tukey test.

In this investigation, the relationships between the expression of defense-related genes and host resistance in treated pea plants with MgO, fungicide, and Zn/MgO against *S. sclerotiorum* were evaluated as presented in Figure 4A–C. Significant fold differences for the expression of selected genes in the treated pea plants with MgO, fungicide, and Zn/MgO compared to the untreated control were identified. The expression of OPR1 increased 3.1, 3.2, and 3.5-fold in MgO, fungicide, and Zn/MgO-treated pea plants under *S. sclerotiorum* infection compared to the untreated plants, respectively (Figure 4A). For the peroxidase (PsOXII) gene, the expression increased 2.7, 2.8, and 3.0-fold in pea plants treated with MgO, fungicide, and Zn/MgO compared to untreated controls under *S. sclerotiorum* infection, respectively (Figure 4B). Additionally, the transcript level of the chlorophyll a-b binding protein (chlorophyll) increased 3.5, 2.8, and 5.0-fold in pea plants treated with MgO, fungicide, and Zn/MgO compared to untreated controls under *S. sclerotiorum* infection, respectively (Figure 4C).

### 3.6. Effect of the Tested Treatments on Free, Conjugated, and Total Phenols in Treated Pea During the Two Tested Seasons (2021–2022 and 2022–2023)

The effects of the investigated nanoparticles and chemical fungicide on free, conjugated, and total phenols are displayed in Table 4 under field conditions. In both growth seasons, the treated pea exhibited a significant (*p* = 0.05) increase in free, conjugated, and total phenols when compared to the untreated control. In treated peas, Zn/MgO had the greatest results for free, conjugated, and total phenol, while MgO and fungicide came in second and third, respectively, in both growth seasons. Under all treatments studied, the measured levels of free, conjugated, and total phenols were higher in the second season than in the first.

### 3.7. Effect of the Tested Treatments on Some Growth Parameters of Pea

Table 5 demonstrate the impact of the chemical fungicide and tested nanoparticles on a few growth indicators (plant height, number of leaves, and fresh and dry weight) during the course of the two testing seasons (2021–2022 and 2022–2023) in the field. In comparison to the untreated control, the tested treatments considerably (*p* = 0.05) improved the pea’s growth characteristics during both growing seasons. Throughout both growing seasons, Zn/MgO nanoparticles had the highest indicators of growth for pea plants, followed by fungicide and MgO, in that sequence. There was a steady rise in every growth parameter that was examined when the tested nanoparticles were applied at higher rates during the two growing seasons. Under all circumstances, the first season’s measured growth characters were higher than those of the second season.

## 4. Discussion

One of the most promising approaches to plant protection is nanotechnology, which aids in the development of alternate management plans for effective disease control in the field [24,27,34]. Furthermore, lowering pesticide contamination in the environment is greatly aided by reducing disease and increasing the production of crops. A promising result of the advancement of nanotechnology is NP-antimicrobial compounds due to their broad spectrum and functionalization properties. The disadvantages of conventional antimicrobial treatments can be eliminated with NP-antimicrobial compounds [54].

In this study, a greenhouse experiment revealed that Zn/MgO outperforms MgO significantly with respect to severity reduction and growth characteristics of pea plants. Metallic nanoparticles have unique physicochemical characteristics and antimicrobial properties due to their high surface area, strong reactivity, and unique particle form. This makes them a great way to overcome microbial resistance [55].

The antifungal activity of two nanoparticles (MgO and Zn/MgO) against *S. sclerotiorum* was assessed in this work. The findings demonstrated that, in laboratory settings, the examined nanoparticles exhibited fungicidal action and reduced the growth of *S. sclerotiorum* at various doses. Promising outcomes were seen when magnesium oxide nanoparticles (MgO-NPs) were studied for the control of phytopathogenic fungi [56,57,58]. Additionally, bimetal oxide nanoparticles (ZnO-MgO) have been shown to exhibit exceptional antifungal characteristics based on their effects on the growth of the phytopathogenic fungus *Colletotrichum gloeosporioides* [59].

Our findings demonstrated that, when compared to untreated controls, the disease severity in pea plants treated with mancozeb, MgO, and Zn/MgO was much lower in greenhouse conditions. The antimicrobial properties of magnesium oxide nanoparticles (NPs) include their low toxicity to human cells, easy availability, and environmental biocompatibility compared to other metal oxides [60,61]. MgO NPs have also been found to function as phytopathogenic antagonists against *Phytophthora parasitica* [57], *Fusarium oxysporum* [62], *Thielaviopsis basicola*, and *Phytophthora parasitica* [57].

The gene analysis showed that pea plants treated with Zn/MgO developed defense mechanisms against *S. sclerotiorum*. These defenses included turning on genes related to stress, making antioxidants like peroxidase, and increasing proteins that help with plant growth. This response is part of a process called systemic acquired resistance (SAR), which enhances the activity of certain genes, such as OPR1, PsOXII, and chlorophyll genes. The gene responses related to plant defense seen in this study match what has been found in earlier research. The increase in the OPR1 gene is linked to resistance in pea plants after infection by *P. koolunga*, affecting both leaves and stems [32,44,63]. In common bean plants, the PR1 gene helps activate signals that may trigger defense responses [64,65,66]. During the interaction between *P. koolunga* and pea plants, higher levels of PsOXII help protect plant tissues by reducing damage caused by oxidative stress during infection [67]. Oxidative stress is important in how plants function because it triggers the production of enzymes that help remove harmful reactive oxygen species (ROS). These enzymes, especially peroxidases, play a key role in breaking down hydrogen peroxide, a type of ROS. This process helps protect plant cells from damage and keeps them healthy, especially when plants face diseases or other stresses [68]. Stronger gene induction of *OPR1, PR1, PsOXII*, and peroxidases is often associated with disease suppression, as these components are vital to the defense signaling and physical reinforcement (e.g., lignin synthesis) pathways. A synergistic relationship between increased antioxidant activity (to manage ROS) and phenolic accumulation (for structural reinforcement) provides superior resistance [69]. Peroxidases perform both managing excess reactive oxygen species (ROS) to inhibit damage to the plant and cross-linking phenolic compounds (like lignin and tannins) into the cell wall. This joined antioxidant-strengthening action, coupled with the jasmonic acid-mediated responses often determined by *OPR1* (oxophytodienoate reductase), improves physical and chemical barriers [70]. Though the gene analysis may approve recognized roles, the synergy between peroxidase activity and phenolic-based structural reinforcement (like lignification) is a significant mechanism for improved disease resistance [71]. Research on how environmental factors affect peroxidase enzyme production shows that stressful conditions increase the activity of enzymes like superoxide dismutase and hydrogen peroxide scavengers. These enzymes help remove excess hydrogen peroxide, which is produced when superoxide dismutase activity rises [72]. In treated pea plants infected with *S. sclerotiorum*, the gene responsible for chlorophyll was more active. Chlorophyll is a protein that captures light for photosynthesis, which is essential for plant growth and development [73]. Finding the genes that help plants resist infections like *S. sclerotiorum* is important for breeding new pea varieties that can better fight the disease.

Plant secondary metabolites, such as phenolic compounds, play an essential role in plant resistance [74]. In this pea study, treatment with MgO and Zn/MgO significantly increased the phenol content compared to the untreated control. This is consistent with research by Sharam et al. [75], who found that chickpeas treated with MgO-NPs exhibited elevated levels of antioxidant markers such as phenolic content. According to these findings, these nanoparticles have been proven as a resistance elicitor for pea plants against white mold. According to Hussain et al. [76], thidiazuron and MgO-NPs have increased the flavonoid and phenolic content of radish. Magnesium (Mg) is considered an essential element for plant growth and development because it has an important role in plant defense against abiotic stressors [77,78]. The resistance of pea plants to pathogens may be generated by the transformation of phenolic chemicals into lignin and suberin, which mechanically reinforce the plant cell wall. The resistance process, mediated by the accumulation of endogenous salicylic acid (SA), a metabolite downstream of the biosynthetic pathway initiated by phenylalanine ammonia-lyase (PAL), is known as systemic acquired resistance (SAR). It is based on the induction of secondary metabolic pathways and the increased synthesis of products, including phenolic compounds, by this metabolism in response to pathogen attack [79].

Our study’s findings demonstrated that, in comparison to the untreated control, pea plants’ growth characteristics were enhanced by treatment with MgO and Zn/MgO. This is in line with the points raised by Abbas et al. [80], who reported that magnesium (Mg) is a nutrient that is vital to plants. It is needed for a number of physiological functions, such as photosynthesis, enzyme function, and general plant growth. Furthermore, magnesium oxide nanoparticles (Mg^+^NPs) have attracted attention in recent agricultural research as a possible way to improve crop yield and plant development [80]. Moreover, exposure to magnesium oxide nanoparticles has been previously shown to enhance the morphological and biochemical characteristics of horse gram, another legume crop [81]. According to the horse gram, MgO-NPs in the current investigation raised the amount of carbohydrates and chlorophyll in black chickpea. Because magnesium is the main component of chlorophyll, it is essential for photosynthesis. Also, MgO-NPs can raise plant Mg content, increasing chlorophyll levels, improving photosynthesis, and activating enzymes essential for protein biosynthesis [82]. These modifications can together enhance plant development [83].

## 5. Conclusions

In both laboratory and greenhouse settings, *S. sclerotiorum* growth and disease severity were dramatically reduced following treatment with the tested nanoparticles as compared to the untreated control. The defense mechanism of pea plants against *S. sclerotiorum* is induced by enhancing the expression of the *OPR1, PsOXII*, and Chlorophyll genes. Phenolic content in pea plants treated with the tested nanoparticles was higher than that of the control. The application of fungicide and tested nanoparticles to peas greatly enhanced their growth properties. The high cost of production and a lack of large-scale, cost-effective manufacturing limit the broad use of nanoproducts for plant disease control, with only a few commercialized nanoproducts currently available. Furthermore, the long-term ecological effects, such as bioaccumulation and impact on food safety, are still relatively unknown.

## Figures and Tables

**Table 1:** Nucleotide sequence Primers used for Quantitative Real-time-PCR analysis of reference and targeted genes.

Abbreviation	Gene	Forward Primer (5–3)	Reverse Primer (3–5)	Accession No.
*OPR1*	12-oxophytodienoate reductase 11	AAGGCTTACCAGACACACCG	GGTTCCACCATGTGGCAGTA	XM_051051558.1
*PsOXII*	peroxidase P7	TTTCTGCTTGGAGGACCCAC	GACTGTTGGCAGCACTTTGG	AB193816.1
Chlorophyll	chlorophyll a-b binding protein 13	TGGCATTGATGGCAGCTACA	ATCGTCGGCAAGACCAAGAG	XM_051027174.1
Alaska alpha-tubulin	House Keeping	GTGCAGAGGGCTGTGTGTAT	AGACAAACGAAAAAGAGCAATGGA	U12589.1

**Table 2:** Radial growth and inhibition percentage of the tested nanoparticles and fungicide against *S. sclerotiorum* in vitro with regression equation and degree of correlation.

Treatment	Concentration (mg ai/L)	Inhibition %	Regression Equation	R^2^
Fungicide	2 4 6 8 10	55 ± 1.1 69 ± 2.1 80 ± 0.74 92 ± 2.45 100 ± 0.0	y = 5.65x + 45.3	0.992
MgO	20 40 60 80 100	52 ± 0.74 65 ± 1.21 75 ± 2.1 91 ± 1.89 100 ± 0.0	y = 0.61x + 40	0.994
Zn/MgO	20 40 60 80 100	57 ± 0.36 69 ± 0.74 77 ± 1.37 92 ± 2.15 100 ± 0.0	y = 0.545x + 46.3	0.991

Each mean value came from three replicates.

**Table 3:** Efficacy of the tested nanoparticles and fungicide against white mold disease in two growing seasons.

Treatments	Conc. (g ai/kg Seeds)	Season 1			Season 2		
		Pre	Post	Survival	Pre	Post	Survival
Zn/MgO	0.080 0.100	10.0 ± 0.52 ^bc^ 7.5 ± 0.25 ^b^	15.0 ± 0.15 ^ab^ 12.5 ± 0.87 ^ab^	75.0 ± 5 ^cd^ 80.0 ± 2.5 ^d^	12.5 ± 1.1 ^ab^ 10.0 ± 0.35 ^ab^	15.0 ± 1.3 ^a^ 12.5 ± 1.1 ^a^	72.5 ± 2.5 ^bc^ 77.5 ± 2.5 ^c^
MgO	0.080 0.100	15.0 ± 0.76 ^c^ 10.0 ± 0.91 ^bc^	20.0 ± 0.97 ^b^ 20.0 ± 1.2 ^b^	65.0 ± 1.5 ^b^ 70.0 ± 2.5 ^bc^	15.0 ± 1.2 ^b^ 12.5 ± 1.3 ^ab^	17.5 ± 087 ^a^ 17.5 ± 1.25 ^a^	67.5 ± 2.12 ^b^ 70.0 ± 2.5 ^b^
Fungicide	1.0	0.0 ^a^	10.0 ± 0.65 ^a^	90.0 ± 3.1 ^e^	5.0 ± 0.4 ^a^	10.0 ± 0.54 ^a^	85.0 ± 2.45 ^d^
control	0.0	35.0 ± 1.23 ^e^	50.0 ± 1.34 ^c^	15.0 ± 0.71 ^a^	32.5 ± 1.34 ^c^	55.0 ± 2.41 ^b^	12.5 ± 0.91 ^a^

Values shown are the means and standard errors (±SE) of four replicates. Statistical comparisons were made among treatments within a single column. The different letters represent significant differences between the means using the Tukey test and the significance threshold (*p* ≤ 0.05).

**Table 4:** Effect of the tested nanoparticles and fungicide on phenol compounds content in pea in two growing seasons.

Treatments	Conc. (g ai/kg Seeds)	Season 1			Season 2		
		Free Phenol (µg/mL)	Conjugated Phenol (µg/mL)	Total Phenol (µg/mL)	Free Phenol (µg/mL)	Conjugated Phenol (µg/mL)	Total Phenol (µg/mL)
Zn/MgO	0.080 0.100	13 ± 1 ^bc^ 15 ± 1 ^c^	19 ± 1 ^cd^ 20 ± 1 ^d^	32 ± 1 ^cd^ 35 ± 2.2 ^d^	12 ± 1 ^cd^ 14 ± 1 ^d^	18 ± 1.5 ^c^ 16 ± 1 ^bc^	30 ± 2 ^c^ 30 ± 2 ^c^
MgO	0.080 0.100	12 ± 1 ^bc^ 10 ± 1 ^b^	14 ± 1 ^b^ 16 ± 1 ^bc^	26 ± 2.1 ^b^ 26 ± 2 ^b^	10 ± 1 ^c^ 7.0 ± 0.25 ^b^	15 ± 1 ^bc^ 14 ± 1 ^b^	25 ± 2 ^b^ 21 ± 1 ^b^
Fungicide	1.0	13 ± 1 ^bc^	18 ± 1 ^cd^	28 ± 2 ^bc^	13 ± 1 ^d^	17 ± 1 ^bc^	30 ± 2 ^c^
Control	--	4.0 ± 0.2 ^a^	6.0 ± 0.3 ^a^	10 ± 0.1 ^a^	3.0 ± 0.2 ^a^	5.0 ± 0.4 ^a^	8.0 ± 0.6 ^a^

Values shown are the means and standard errors (±SE) of four replicates. Statistical comparisons were made among treatments within a single column. The different letters represent significant differences between the means using the Tukey test and the significance threshold (*p* ≤ 0.05).

**Table 5:** Effect of the tested nanoparticles and fungicide on some growth characters of pea plants in two growing seasons.

Treatments	Conc. (g ai/kg Seeds)	Season 1				Season 2			
		Plant Height (cm)	No. of Leaves	Fresh Weight (g)	Dry Weight (g)	Plant Height (cm)	No. of Leaves	Fresh Weight (g)	Dry Weight (g)
Zn/MgO	0.080 0.100	43 ± 1 ^d^ 45 ± 1 ^d^	27 ± 2 ^bc^ 30 ± 1 ^cd^	38 ± 1 ^cd^ 40 ± 1 ^d^	2.4 ± 0.2 ^d^ 2.2 ± 0.2 ^cd^	40 ± 2 ^cd^ 42 ± 2 ^d^	25 ± 1 ^b^ 28 ± 2 ^bc^	37 ± 2 ^d^ 39 ± 1 ^d^	2.3 ± 0.2 ^c^ 2.0 ± 0.1 ^bc^
MgO	0.080 0.100	32 ± 2 ^b^ 37 ± 2 ^c^	24 ± 1 ^b^ 26 ± 2 ^b^	30 ± 1 ^b^ 35 ± 2 ^c^	1.6 ± 0.2 ^b^ 1.8 ± 0.1 ^bc^	30 ± 2 ^b^ 36 ± 2 ^c^	22 ± 1 ^ab^ 23 ± 2 ^ab^	28 ± 2 ^b^ 32 ± 1 ^c^	1.6 ± 0.2 ^b^ 1.8 ± 0.1 ^b^
Fungicide	1.0	44 ± 1 ^d^	32 ± 2 ^d^	37 ± 2 ^cd^	1.9 ± 0.1 ^bc^	39 ± 1 ^cd^	32 ± 2 ^c^	38 ± 1 ^d^	1.7 ± 0.1 ^b^
Control	-	25 ± 1 ^a^	20 ± 1 ^a^	22 ± 1 ^a^	1.1 ± 0.1 ^a^	23 ±1 ^a^	17 ± 1 ^a^	20 ± 1 ^a^	1.0 ± 0.1 ^a^

Values shown are the means and standard errors (±SE) of four replicates. Statistical comparisons were made among treatments within a single column. The different letters represent significant differences between the means using the Tukey test and the significance threshold (*p* ≤ 0.05).

## Data Availability

The original contributions presented in the study are included in the article, further inquiries can be directed to the corresponding authors.

## References

[1] Dong D., Xiang C. (2021). Three Sclerotinia species as the cause of white mold on pea in Chongqing and Sichuan of China. J. Integr. Agric..

[2] FAO (2012). Food and Agricultural Organization of the United Nations.

[3] Bhat T.A., Gupta M., Ganai M.A., Ahanger R.A., Bhat H.A. (2013). Yield, soil health and nutrient utilization of field pea (*Pisum sativum* L.) as affected by phosphorus and biofertilizers under subtropical conditions of Jammu. Inter. J. Mod. Plant Anim. Sci..

[4] Ceyhan E., Avci M.A. (2005). Combining ability and heterosis for grain yield and some yield components in pea (*Pisum sativum* L.). Pak. J. Biol. Sci..

[5] Makasheva R.K. (1983). The Pea.

[6] Haque S.R., Akter N., Khan M.A.H., Kabir K., Islam M.M. (2014). Yield potential of garden pea varieties at varied harvesting dates. Bangladesh Agronmy J..

[7] Bolland G.J., Hall R. (1994). Index of plant hosts of *Sclerotinia sclerotiorum*. Can. J. Plant Pathol..

[8] Bolton M.D., Thomma B.P.H.J., Nelson B.D. (2006). *Sclerotinia sclerotiorum* (Lib.) de Bary: Biology and molecular traits of a cosmopolitan pathogen. Mol. Plant Pathol..

[9] Liang X., Rollins J.A. (2018). Mechanisms of broad host range necrotrophic pathogenesis in *Sclerotinia sclerotiorum*. Phytopathology.

[10] Huang H.C., Erickson R.S. (2000). Soil treatment with fungal agents for control of apothecia of *Sclerotinia sclerotiorum* in bean and pea crops. Plant Pathol. Bull..

[11] Kim W.G., Hong S.K., Lee S.Y. (2006). Occurrence of *Sclerotinia rotinfour* leguminous crops caused by *Sclerotinia sclerotiorum*. Plant Pathol. J..

[12] Smolinska U., Kowalska B. (2006). Biological control of the soil-borne fungal pathogen *Sclerotinia sclerotiorum*―A review. J. Plant Pathol..

[13] Wang S.Y., Zhang Y.J., Chen X., Shi X.C., Herrera-Balandrano D.D., Liu F.Q., Laborda P. (2024). Biocontrol methods for the management of *Sclerotinia sclerotiorum* in legumes: A Review. Phytopathology.

[14] Kabbage M., Yarden O., Dickman M.B. (2015). Pathogenic attributes of *Sclerotinia sclerotiorum:* Switching from a biotrophic to necrotrophic lifestyle. Plant Sci..

[15] Singh M., Avtar R., Kumar N., Punia R., Lakra N., Kumari N., Bishnoi M., Rohit R., Choudhary R.R., Khedwal R.S. (2023). Assessment of Sclerotinia Stem and Leaf Rot Resistance and its Association with Physical Strength Attributes in Brassicaceae with Special Emphasis on *Brassica Juncea*. J. Plant Growth Regul..

[16] Shahoveisi F., Del Rio Mendoza L.E. (2020). Effect of wetness duration and incubation temperature on development of ascosporic infections by *Sclerotinia sclerotiorum*. Plant Dis..

[17] Antwi-Boasiako A., Wang Y., Dapaah H.K., Zhao T.J. (2022). Mitigating against Sclerotinia diseases in legume crops: A comprehensive review. Agronomy.

[18] Mazumdar P. (2021). Sclerotinia stem rot in tomato: A review on biology, pathogenicity, disease management and future research priorities. J. Plant Dis. Prot..

[19] Ajiboye T.O., Visser H.G., Erasmus E., Schutte-Smith M. (2025). Recent strategies for controlling Phytoparasitica, 37al pathogen (*Sclerotinia sclerotiorum*) using gene silencing, botanical fungicides and nanomaterials. Sustain. Food Technol..

[20] Ma H.X., Chen Y., Wang J.X., Yu W.Y., Tang Z.H., Chen C.J., Zhou M.G. (2009). Activity of carbendazim, dimethachlon, iprodione, procymidone and boscalid against Sclerotinia stem rot in Jiangsu Province of China. Phytoparasitica.

[21] Hazra R.S., Roy J., Jiang L., Webster D.C., Rahman M.M., Quadir M. (2023). Biobased, macro-, and nanoscale fungicide delivery approaches for plant fungi control. ACS Appl. Bio. Mater..

[22] Pestovsky Y.S., Martínez-Antonio A. (2017). The use of nanoparticles and nanoformulations in agriculture. J. Nanosci. Nanotechnol..

[23] Derbalah A., Shenashen M., Hamza A., Mohamed A., El Safty S. (2018). Antifungal activity of fabricated mesoporous silica nanoparticles against early blight of tomato. Egypt. J. Basic. Appl. Sci..

[24] Derbalah A., Elsharkawy M., Hamza A., El-Shaer A. (2019). Resistance induction in cucumber and direct antifungal activity of zirconium oxide nanoparticles against *Rhizoctonia solani*. Pestic. Biochem. Physiol..

[25] Derbalah A.S., Elsharkawy M.M. (2019). A new strategy to control Cucumber mosaic virus using fabricated NiO-nanostructures. J. Biotechnol..

[26] Elsharakawy M., Derbalah A., Hamza A., El-Shaer A. (2020). Zinc oxide nanostructures as a control strategy of bacterial speck of tomato caused by *Pseudomonas syringae* in Egypt. Environ. Sci. Pollut. Res..

[27] Kamel S.M., Elgobashy S.F., Omara R.I., Derbalah A.S., Abdelfatah M., El-Shaer A., Al-Askar A.A., Abdelkhalek A., Abd-Elsalam K.A., Essa T. (2022). Antifungal activity of copper oxide nanoparticles against root rot disease in cucumber. J. Fungi.

[28] Boxi S.S., Mukherjee K., Paria S. (2016). Ag doped hollow TiO_2_ nanoparticles as an effective green fungicide against *Fusarium solani* and *Venturia inaequalis* phytopathogens. Nanotechnology.

[29] Chen J., Wang X., Han H. (2013). A new function of graphene oxide emerges: Inactivating phytopathogenic bacterium, *Xanthomonas Oryzae pv. oryzae*. J. Nanopart. Res..

[30] Shah M.A., Towkeer A. (2010). Principles of Nanosciences and Nanotechnology.

[31] Chauhan D., Kumar R., Thakur N., Singh M., Kumar K. (2024). *Ocimum sanctum*-mediated co/cu/zn-doped magnesium oxide nanoparticles: Photocatalytic, antibacterial, and antioxidant properties for environmental remediation. Hybrid. Adv..

[32] Fondevilla S., Kuster H., Krajinski F., Cubero J.I., Rubiales D. (2011). Identification of genes differentially expressed in a resistant reaction to *Mycosphaerella pinodes* in pea using microarray technology. BMC Genom..

[33] Ma Y., Wang L., Cao Z., Wang H., Wang F., Zhu W. (2025). SlMYC2 Mediates the JA pathway by responding to chlorocholine chloride in the regulation of resistance to TYLCD. Plants.

[34] Derbalah A., Essa T., Kamel S.M., Omara R.I., Abdelfatah M., Elshaer A., Elsharkawy M.M. (2022). Silver oxide nanostructures as a new trend to control strawberry charcoal rot induced by *Macrophomina phaseolina*. Pest Manag. Sci..

[35] Khanday A.H., Badroo I.A., Wagay N.A., Rafiq S. (2024). Role of phenolic compounds in disease resistance to plants. Plant Phenolics in Biotic Stress Management.

[36] Cheynier V., Comte G., Davies K.M., Lattanzio V., Martens S. (2013). Plant phenolics: Recent advances on their biosynthesis, genetics, and ecophysiology. Plant Physiol. Biochem..

[37] Lattanzio V., Lattanzio V.M.T., Cardinali A., Imperato F. (2006). Role of phenolics in the resistance mechanisms of plants against fungal pathogens and insects. Phytochemistry Advances in Research.

[38] Treutter D. (2005). Significance of flavonoids in plant resistance and enhancement of their biosynthesis. Plant Biol..

[39] Farkas G.L., Kiraly Z. (1962). Role of phenolic compounds in the physiology of plant diseases and disease resistance. J. Phytopathol..

[40] He Y.S., Ingudam S., Reed A., Gehring T.P., Strobaugh P.I. (2016). Study on the mechanism of antibacterial action of magnesium oxide nanoparticles against foodborne pathogens. J. Nanobiotechnol..

[41] El-Shaer A., Abdelfatah M., Mahmoud K.R., Momay S., Eraky M.R. (2020). Correlation between photoluminescence and positron annihilation lifetime spectroscopy to characterize defects in calcined MgO nanoparticles as a first step to explain antibacterial activity. J. Alloys Comp..

[42] Sundrarajan M., Suresh J., Gandhi R.R. (2012). A comparative study on antibacterial properties of MgO nanoparticles prepared under different calcination temperature. Dig. J. Nanomater. Biostruct..

[43] Vincent J.M. (1947). Distortion of fungal hyphae in the presence of certain inhibitors. Nature.

[44] Tran H.S., You M.P., Barbetti M.J. (2018). Expression of defence-related genes in stems and leaves of resistant and susceptible field pea (*Pisum sativum*) during infection by *Phoma koolunga*. Plant Pathol..

[45] Gomez K.A., Gomez A.A. (1984). Statistical Procedures for Agricultural Research.

[46] Snell F.D., Snell C.T. (1953). Colorimetric Methods of Analysis, Including Some Turbidimetric and Nephelometric Methods.

[47] Boudhrioua N., Bahloul N., Slimen I.B., Kechaou N. (2009). Comparison on the total phenol contents and the color of fresh and infrared dried olive leaves. Indust. Crops Prod..

[48] El-Gali Z.I. (2015). Influence of seeds and roots extracts and exudates of bean plant on growth of some pathogenic fungi. Open Access Libr. J..

[49] Ding Y., Zhang G., Wu H., Hai B., Wang L., Qian Y. (2001). Nanoscale magnesium hydroxide and magnesium oxide powders: Control over size, shape, and structure via hydrothermal synthesis. Chem. Mater..

[50] Mirzaei H., Davoodnia A. (2012). Microwave assisted sol-gel synthesis of MgO nanoparticles and their catalytic activity in the synthesis of hantzsch 1, 4-dihydropyridines. Chin. J. Catal..

[51] Pashchanka M., Hoffmann R.C., Schneider J.J. (2020). Controlled synthesis and characterization of MgO nanoparticles, thin films and polycrystalline nanorods derived from a Mg (ii) single source precurso. J. Mater. Chem..

[52] Wang D., Chen Z.Q., Wang D.D., Qi N., Gong J., Cao C.Y., Tang Z. (2010). Positron annihilation study of the interfacial defects in ZnO nanocrystals: Correlation with ferromagnetism. J. Appl. Phys..

[53] Sayed F.A., Eissa N.G., Shen Y., Hunstad D.A., Wooley K.L., Elsabahy M. (2022). Morphologic design of nanostructures for enhanced antimicrobial activity. J. Nanobiotechnol..

[54] Hemeg H.A. (2017). Nanomaterials for alternative antibacterial therapy. Inter. J. Nanomed..

[55] Wang L., Hu C., Shao L. (2017). The antimicrobial activity of nanoparticles: Present situation and prospects for the future. Inter. J. Nanomed..

[56] Wani A.H., Amin M., Shahnaz M., Shah M.A. (2012). Antimycotic activity of nanoparticles of MgO, FeO and ZnO on some pathogenic fungi. Inter. J. Manufact Mater. Mech. Engin..

[57] Chen J., Wu L., Lu M., Lu S., Li Z.Y., Ding W. (2020). Comparative study on the fungicidal activity of metallic MgO nanoparticles and macroscale MgO against soilborne fungal phytopathogens. Front. Microbiol..

[58] Sidhu A., Bala A., Singh H., Ahuja R., Kumar A. (2020). Development of MgO-sepoilite Nanocomposites against Phytopathogenic Fungi of Rice (Oryzae sativa): A Green Approach. ACS Omega.

[59] De la Rosa-García S.C., Martínez-Torres P., Gómez-Cornelio S., Corral-Aguado M.A., Quintana P., Gómez-Ortíz N.M. (2018). Antifungal activity of ZnO and MgO nanomaterials and their mixtures against *Colletotrichum gloeosporioides* strains from tropical fruit. J. Nanomater..

[60] Wang Z.L., Zhang X., Fan G.J., Que Y., Xue F., Liu Y.H. (2022). Toxicity effects and mechanisms of MgO nanoparticles on the oomycete pathogen Phytophthora infestans and its host *Solanum tuberosum*. Toxics.

[61] Bagheri M., Roshanaei G., Asgari G., Chavoshi S., Ghasemi M.J.D., Treatment W. (2020). Application of carbon-doped nanomagnesium oxide for catalytic ozonation of real textile wastewater: Fractional factorial design and optimization. Desalination Wat. Treat..

[62] Parizi M.A., Moradpour Y., Roostaei A., Khani M., Negahdari M., Rahimi G. (2014). Evaluation of the antifungal effect of magnesium oxide nanoparticles on Fusarium oxysporum F. Sp. lycopersici, pathogenic agent of tomato. Europ. J. Exp. Biol..

[63] Wilson S.K., Pretorius T., Naidoo S. (2023). Mechanisms of systemic resistance to pathogen infection in plants and their potential application in forestry. BMC Plant Biol..

[64] Borges A., Melotto M., Tsai S., Caldas D. (2012). Changes in spatial and temporal gene expression during incompatible interaction between common bean and anthracnose pathogen. J. Plant Physiol..

[65] Mostafa A.A., El-Rahman S.N., Shehata S.N., Naglaa A.A., Hanaa S.O. (2020). Assessing the effects of a novel biostimulant to enhance leafminer resistance and plant growth on common bean. Sci. Rep..

[66] Omar H.S., Hagag M.H., El-Khishin D., Hashem M. (2025). Expression of pathogenesis-related proteins in potato brown rot plants confers resistance to filamentous pathogens under field trials. Sci. Rep..

[67] Joshi S., Pandey B.R., Rosewarne G. (2022). Characterization of field pea (*Pisum sativum*) resistance against *Peyronellaea pinodes* and *Didymella pinodella* that cause ascochyta blight. Front. Plant Sci..

[68] Sood M. (2025). Reactive oxygen species (ROS): Plant perspectives on oxidative signalling and biotic stress response. Discov. Plants.

[69] Yang T., Zhang P., Pan J., Amanullah S., Luan F., Han W., Liu H., Wang X. (2022). Genome-wide analysis of the peroxidase gene family and verification of lignin synthesis-related genes in Watermelon. Inter. J. Mol. Sci..

[70] Cai G., Zhang Y., Huang L., Wang N. (2023). Uncovering the role of PdePrx12 peroxidase in enhancing disease resistance in poplar trees. J. Fungi.

[71] Zhang L., Jiang Y., Jin T., Zheng M., Yap Y., Min X., Chen J., Yuan L., He F., Zhou B. (2025). Identification of key biomarkers related to lipid metabolism in acute pancreatitis and their regulatory mechanisms based on bioinformatics and machine learning. Biomedicines.

[72] Kunos V., Cséplő M., Seress D., Eser A., Kende Z., Uhrin A., Mészáros K. (2022). The stimulation of superoxide dismutase enzyme activity and its relation with the *pyrenophora teres f. teres* infection in different barley genotypes. Sustainability.

[73] Sherin G., Aswathi K.R., Puthur J.T. (2022). Photosynthetic functions in plants subjected to stresses are positively influenced by priming. Plant Stress.

[74] Ruiz-García Y., Gómez-Plaza E. (2013). Elicitors: A tool for improving fruit phenolic content. Agriculture.

[75] Sharma P., Gautam A., Kumar V., Guleria P. (2022). MgO nanoparticles priming promoted the growth of black chickpea. J. Agric. Food Res..

[76] Hussain F., Hadi F., Akbar F. (2019). Magnesium oxide nanoparticles and thidiazuron enhance lead phytoaccumulation and antioxidative response in *Raphanus sativus* L. Environ. Sci. Pollut. Res..

[77] Cakmak I. (2013). Magnesium in crop production, food quality and human health. Plant Soil.

[78] Mengutay M., Ceylan Y., Kutman U.B., Cakmak I. (2013). Adequate magnesium nutrition mitigates adverse effects of heat stress on maize and wheat. Plant Soil.

[79] Iriti M., Rossoni M., Borgo M., Ferrara L., Faoro F. (2005). Induction of resistance to gray mold with benzothiadiazole modifies amino acid profile and increases proanthocyanidins in grape: Primary versus secondary metabolism. J. Agric. Food Chem..

[80] Abbas Z., Hassan M.A., Huang W., Yu H., Xu M., Chang X., Liu L. (2024). Influence of magnesium oxide (MgO) nanoparticles on Maize (*Zea mays* L.). Agronomy.

[81] Sharma P., Gautam A., Kumar V., Guleria P. (2021). In vitro exposed magnesium oxide nanoparticles enhanced the growth of legume *Macrotyloma uniflorum*. Environ. Sci. Pollut. Res..

[82] Gerendás J., Führs H. (2013). The significance of magnesium for crop quality. Plant Soil.

[83] Cai L., Chen J., Liu Z., Wang H., Yang H., Ding W.J.F.I.M. (2018). Magnesium Oxide Nanoparticles: Effective agricultural antibacterial agent against *Ralstonia solanacearum*. Front. Microbiol..

